# How Can ‘Health in All Policies’ Help Maximise the Potential of Microbial Biotechnologies for Health, Equity and Sustainability?

**DOI:** 10.1111/1751-7915.70194

**Published:** 2025-07-08

**Authors:** Margaret J. Douglas, Liz Green, James Timmis, Timo Clemens, Kenneth Timmis

**Affiliations:** ^1^ Public Health Scotland, Gyle Square, 1 South Gyle Crescent Edinburgh UK; ^2^ School of Health & Wellbeing, 90 Byres Road University of Glasgow UK; ^3^ Department of International Health, Care and Public Health Research Institute – CAPHRI Maastricht University Maastricht the Netherlands; ^4^ Policy and International Health WHO Collaborating Centre on ‘Investment in Health and Well‐Being’, Public Health Wales Cardiff UK; ^5^ Department of Political Science University of Freiburg Freiburg Germany; ^6^ Athena Institute for Research on Innovation and Communication in Health and Life Sciences Vrije Universiteit Amsterdam Amsterdam the Netherlands; ^7^ Institute of Microbiology Technical University Braunschweig Braunschweig Germany

**Keywords:** governance, health impact assessment, health in all policies, microbial biotechnology, societal impact

## Abstract

Microbial biotechnologies could affect health through multiple pathways, including impacts on food, nutrition, and the physical, economic, and social environment. Health in All Policies is an approach to ensure that plans and policies in all sectors maximise health gains and minimise any health risks. This approach often uses health impact assessment as a structured process to identify and assess positive and negative health impacts and make recommendations to improve these. There are very few examples where HIA has been applied to the implementation of microbial biotechnologies. As more biotechnologies are developed and implemented, more routine use of HIA could help to avoid adverse effects and realise their potential to improve health and reduce health inequalities. This will require greater awareness and understanding of the breadth of links to health, research to build the evidence base for these links, and governance mechanisms to oversee the development and implementation of microbial biotechnologies that prioritise health, equity and sustainability.

## Graphical abstract

Applying Health in All Policies to the development and application of microbial biotechnologies can identify and address a range of possible health determinants.

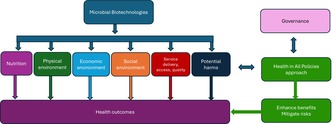

## 1 Introduction

The past two centuries have seen impressive gains in human health—reflected not least in mortality data showing that global life expectancy at birth rose from just 26 years in 1820 to 73 years in 2020 (O'Neill 2024). Yet there remains a high burden of avoidable morbidity and mortality, with significant inequalities within and between countries. For example, life expectancy at birth ranges from 51 years in Lesotho to 84 years in Japan (World Health Organisation 2025a). There are even starker inequalities in child mortality, which ranges from 7 per 1000 in Europe to 70 per 1000 in Africa (World Health Organisation 2025b). This special issue focuses on the third United Nations Sustainable Development Goal (SDG) SDG3, to improve health for all (United Nations 2025). SDG3 sets specific targets for maternal and child mortality, communicable and non‐communicable diseases, road injuries, deaths from pollution and universal healthcare (United Nations 2025). In considering how the SDGs can promote health, it is also notable that the other SDGs include many determinants of health that underpin population health outcomes (Morton et al. 2019). This means that significant progress across all the SDGs is needed to improve health and reduce health inequalities.

Microbial biotechnologies are technologies based upon microbes, their components and products, or their activities. Such technologies can help to achieve SDG3 and many of the other SDGs, and this special issue includes a host of examples of these contributions. Healthcare applications include vaccines (Oxford Vaccine Group 2025), diagnostics (Capin et al. 2024; Chang et al. 2021; Courbet et al. 2015; Zhong et al. 2024), antibiotics (Hutchings et al. 2019) and a wealth of other microbially‐inspired drugs and therapies, including their delivery and microbiome interventions (Alexander et al. 2017; Garmendia and Cebollero‐Rivas 2024) and probiotics (Chua et al. 2017; Gulliver et al. 2022; Vargason and Anselmo 2018). Microorganisms produce many health promoting compounds that could be harnessed to prevent and treat a wide range of conditions. Microbial biotechnologies can also support basic needs for nutrition (Bernal 2024; Diaz‐Troya and Huertas 2024; Graham and Ledesma‐Amaro 2023; Javourez et al. 2024), clean drinking water and sanitation (Lovett 2024), and energy (Liu et al. 2023). All of these bring potential to benefit health, equity and sustainability. Importantly, microbial biotechnologies have huge potential for increasing health resilience in the face of challenges such as global warming (Onyeaka and Ekwebelem 2023) and environmental pollutant removal (Ding et al. 2024; Kariyawasam et al. 2024). Microbiome interventions hold much promise also in relation to mental health (Geirnaert et al. 2017; Gonzales‐Luna et al. 2023). However, the realised health benefits may vary depending on how these biotechnologies are applied and how they are distributed. There is a need for approaches to ensure they are developed and adopted in ways that maximise their benefits, minimise harms and help to reduce health inequalities and inequity globally.

This paper will consider how a Health in All Policies (HiAP) approach could help maximise benefits to health from the use of microbial biotechnologies. We will discuss what HiAP is and focus on the use of health impact assessment (HIA) in a HiAP approach. We will set out some of the health determinants routinely considered in HIAs and how they may be influenced by microbial biotechnologies. We will then discuss some examples showing how HIA could be applied to improve the outcomes of biotechnology implementation or adoption, and how an understanding of microbial biotechnology may inform the practice of HIA.

## 2 Health in All Policies

The World Health Organization defines HiAP as ‘an approach to public policies across sectors that systematically takes into account the health and health systems implications of decisions, seeks synergies and avoids harmful health impacts, in order to improve population health and health equity’ (World Health Organization 2014). This recognises that health is the outcome of interacting actions across sectors, not solely the actions of the healthcare sector. HiAP promotes shared governance for these outcomes. It can be applied to proposed legislation, policies, strategies, and plans at all levels (Green et al. 2021).

Several tools, structures and processes can be used to support HiAP. These include cross sectoral committees, teams or partnerships, which can build shared understanding of links to health and ensure shared accountability for health outcomes (Leppo et al. 2013; World Health Organization 2015). Other tools to support HiAP include shared budgets, joint workforce development and ways to build wider participation such as citizens' juries (WHO and the Government of South Australia 2010). HIA is a process that is specifically designed to identify and influence the health consequences of policies and plans in any sector (Winkler et al. 2021). HIA is a structured, participatory, evidence‐based process. It aims to understand the likely consequences for health of a plan or policy in any sector, to inform changes that maximise health gains and prevent or mitigate any risks to health, and so is particularly useful for implementing HiAP (Green et al. 2021). The steps taken in an HIA process are shown in Table 1. This ‘step by step’ HIA process should be differentiated from a risk assessment that quantifies the toxicity of a hazard, although these are sometimes also called HIA in the literature (Kim et al. 2024).

**TABLE 1:** Steps in an HIA process.

Step	Tasks
Screening	Decide if an HIA is likely to be appropriate and useful
Scoping	Define scope of the HIA, including impacts to assess, methods to use, spatial, time and population boundaries of the HIA
Appraisal	Collect and appraise evidence to assess the impacts on health
Recommendations	Make recommendations for amendments to the proposal to enhance positive and prevent or mitigate negative impacts
Reporting	Produce and share report of findings
Monitoring and evaluation	Monitor implementation of recommendations and impacts that arise after the proposal is implemented

HIA takes a comprehensive approach by systematically considering a range of possible determinants and different populations that a policy or plan may affect (Winkler et al. 2021). This often involves a stakeholder workshop during the screening or scoping step, in which participants use a checklist to prompt consideration of populations and determinants (Public Health Scotland 2024). The workshop discussion is used to identify determinants that may be affected by the proposal. Table 2 shows examples of the populations and determinants included in an HIA checklist used in Scotland (Public Health Scotland 2024). Other HIAs have used the SDGs as a framework to identify impacts (Green et al. 2020; Winkler et al. 2020). The appraisal step of HIA involves using multiple sources of evidence to assess these potential health consequences to inform recommendations for changes to mitigate and improve these impacts. Participation is a key value in HIA (den Broeder et al. 2017). HIAs involve affected communities and stakeholders, often including qualitative evidence of their views and values. This is then used along with other forms of evidence. They promote health equity by assessing differential impacts across populations and prioritising recommendations that will improve health for populations with the poorest health outcomes (Bourcier et al. 2016; Heller, Givens, et al. 2014). HIAs also support sustainability. There is a large overlap between the SDGs and the health determinants considered in HIA, and they assess future, not just current, impacts on health (McDermott et al. 2024; Winkler et al. 2021).

**TABLE 2:** Populations and determinants considered in HIA.

Section	Prompts in HIA checklist
Populations	People of different ages—older people, children and young people, working age adults Men and women Disabled people Different ethnic groups Low‐income people Carers Minority groups People in different geographical areas
Social environment	Social status Stigma Culture, community connectedness and cohesion Stress, uncertainty, resilience and community assets Family and social relationships support Participation and social interaction Influence and sense of control Inclusion, identity and belonging Sense of purpose Public safety, crime and fear of crime
Physical environment	Housing provision, mix, living conditions, security of tenure Local amenities—retail, community venues Public spaces quality and maintenance—litter, fly tipping, dog fouling Natural space and biodiversity Environmental pollution—quality of air, water, soil, light, noise and odours Energy, resource use and waste Greenhouse gas emissions Climate resilience and adaptation—flood protection, coastal erosion, extreme weather Unintentional injuries Transmission of infectious diseases
Economic environment	Access to/levels of employment (paid or unpaid), unemployment and economic inactivity Quality of employment and working conditions—including health and safety, job control, job strain, employee voice, opportunity, security, fulfilment and respect Income and wealth inequality Wealth circulation and community benefits Income security and debt Cost of living Ownership of assets Commercial practices
Access to and quality of services	Health and social care services Housing and homelessness services Transport—available, reliable, affordable, accessible, safe active and public transport Education provision—learning, training, skills development, digital literacy Community venues, culture, leisure and play provision Digital connectivity—access to IT, internet and digital services Environmental and regulatory standards and services Other service provision—e.g. legal, refuse
Commercial influences	Food and nutrition Exercise and physical activity Substance use—tobacco (smoking/vaping), alcohol, drugs Gambling Sexual health Social media use Sleep
Equality between groups	Discrimination, harassment and victimisation Advancing equality of opportunity between groups Promoting good relations between different groups

## 3 Microbial Biotechnologies and Health Determinants

Microbial biotechnologies may affect many of the health determinants routinely considered in HIA, for example, food, safety, the physical environment and working conditions. A few non‐comprehensive examples of these links are discussed briefly below to illustrate this, which include both direct and indirect effects. An HIA workshop of a proposal to introduce or use any biotechnology would aim to identify the range of potential impacts in the screening or scoping step of the HIA to allow these to be included in the assessment (Public Health Scotland 2024). Stakeholders involved in the workshops should have a range of perspectives and may include users and developers of the relevant biotechnology, regulators and others. The workshops identify populations that may be affected in different ways—for example, users or customers of a biotechnology, local communities, workforce, or others in a supply chain. The impacts are likely to vary between these populations, and within each of these groups, there could also be differences by personal characteristics such as age, sex or disability. It is important to recognise these differences to ensure potential positive or negative impacts on health equity are identified and addressed—either by enhancing positive impacts or mitigating negative ones (Public Health Scotland 2024).

### 3.1 Food and Nutrition

An estimated 1 in 11 people globally were affected by hunger in 2023 and in 2022 an estimated 29% of the world population could not afford a healthy diet (FAO, IFAD, UNICEF, WFP and WHO 2024). This results not only from insufficient availability—quantity and distribution—but also insufficient quality, such as micronutrient deficiency and ultraprocessing (Timmis et al. 2025). Microbial foods (microbial biomass and fermented foods) and microbial flavourings and condiments have high nutritional value and some may increase the diversity of the gut microbiome (García et al. 2017; Kiczorowski et al. 2022; Sawant et al. 2025). Fermented foods also have increased shelf life, thereby reducing waste (Jahn et al. 2023). Other biotechnologies including animal probiotics, agrobiochemicals including plant inocula, ensilaging plant materials used for animal feed, vaccines and microbially‐inspired therapeutic agents can also increase food availability and help to reduce hunger and improve fuller nutrition (Guo et al. 2023). However, these health benefits depend on factors including how biotechnologies are used, how they are distributed, and the financial and other costs of their introduction and use.

### 3.2 Physical Environment

The physical environment is an important determinant of health. Environmental pollution of air, water, and land causes approximately 9 million deaths per year, or 1 in 6 deaths globally (Fuller et al. 2022). In 2022, 2.2 billion people lacked access to clean water, which is a pre‐requisite for health, and 1.5 billion lack basic sanitation (United Nations 2023). Microbes are chemical transformers *par excellence* and responsible for the degradation of almost all wastes of biological origin and many industrial pollutants. Wastewater treatment and pathogen removal is a microbial biotechnology of varying sophistication that can be applied essentially everywhere. Drinking water preparation involves microbial biotechnology, as do a wide range of mitigation processes for polluted air, water and land (Timmis et al. 2022). Urban overheating, especially in densely built urban environments (heat islands) is an important cause of morbidity and mortality due to heat stress and pollution, which particularly affects occupants of low‐income housing and cities in LMICs, with inhabitants of informal settlements and refugee camps particularly vulnerable (Santamouris 2020). While the evidence base is still limited, novel approaches using microalgae to reduce heat stress or capture carbon as part of building facades and/or in built urban environments are being tested (in vivo or in silico) and the results are encouraging (Cervera et al. 2024; Pozzobon 2024). The physical environment can also promote good health—for example, exposure to greenspace has positive impacts on multiple health outcomes (Egorov et al. 2024; Xie et al. 2024). However, as with nutritional applications, the impacts on health and health equity depend on how they are used and the extent to which people in most need can access these benefits.

### 3.3 Economic Environment

Microbial biotechnologies are also likely to have economic effects, which could be substantial (Timmis et al. 2017). The development and production of biotechnologies may generate new employment, and/or may displace other industries and reduce jobs elsewhere. Good quality employment is a strong determinant of health, and long‐term unemployment has substantial negative impacts on physical and mental health (Paul and Moser 2009; Roelfs et al. 2011; Thomson et al. 2023). This raises questions about how biotechnologies will be introduced, whether they will provide high‐quality, well‐paid employment and, if so, who will benefit from these jobs. Biotechnologies can be used to generate large profits (Timmis et al. 2017). If they can be licenced and used by small community enterprises, this may benefit local economies (McInroy 2018). However, if the rights to exploit biotechnologies are restricted to a few large corporations, this could further increase the global and national concentrations of income and wealth that continue to underpin health inequalities (Wilkinson and Pickett 2009).

### 3.4 Social Environment

There could also be social impacts. Technological innovations, such as biotechnologies relating to the production of microbial food or to control pests, may challenge traditional cultures or practices (Alsaleh 2024). If industrial development of biofuels or other biotechnologies involves the migration of workers or displacement of people, there could be crowding and impacts on social and family relationships (Greene et al. 2011). This could be associated with a range of potential adverse psychosocial effects, such as increased stress, social exclusion, lack of social support, and further consequences such as mental health issues, anomie, and/or addiction (Wilkinson and Marmot 2003).

### 3.5 Service Delivery, Access and Quality

Some microbial biotechnologies may influence the delivery of services, including healthcare, transport and housing. These include medical technologies such as vaccination or diagnostics and biofuels for transport or heating. For each of these, there should be consideration of the infrastructure needed to ensure equitable access to these and any wider impacts. For example, changes in service delivery may have environmental impacts related to management of waste or patient or staff travel (Lenzen et al. 2020) and may affect working conditions for relevant staff (Backhaus et al. 2024). Introduction of new biotechnologies may also either raise or lower costs (Konzock and Nielsen 2024), which would particularly affect low‐income consumers.

These are just some examples of the potential links between microbial biotechnologies and a range of health determinants. Taking a Health in All Policies approach to the introduction of biotechnologies could ensure they were directed in ways that enhance the positives, mitigate the risks to health, and make a positive contribution to health equity.

## 4 Applying HiAP to Microbial Biotechnologies

The previous section discussed just a few of the individual determinants that microbial biotechnologies may influence. A key principle of HiAP is that it should involve a comprehensive consideration of a range of potential determinants and health outcomes (Green et al. 2021). We are aware of relatively few examples of HIAs conducted on microbial biotechnologies. However, here we present two illustrative examples of the range of impacts that relevant developments may have.

### 4.1 Commercial Production of Biofuels

Microbial biotechnology can contribute to energy security and reduce greenhouse gas emissions by converting plant material and waste into biofuels (Love 2022). Commercial developments producing biofuels could have significant economic, social, and health impacts for communities where they are located, especially in low‐income settings. This is illustrated by a combined Environmental, Social, and Health Impact Assessment (ESHIA) that was conducted on a biofuel development in a rural area of Sierra Leone with around 13,000 inhabitants (Manley et al. 2011; Winkler et al. 2014). The ESHIA was guided by national and international legislative requirements for environmental and social management (International Finance Corporation 2009). The environmental and social impact sections of the combined report identified many impacts relevant for health (Manley et al. 2011). The HIA included a survey to assess the baseline health status as well as drawing on routine data and stakeholder consultation (Winkler et al. 2014). Table 3 shows the range of health impacts identified in the HIA. The HIA informed a health and safety management plan with measures to mitigate adverse impacts. These included management of occupational and traffic risks and a Farmer Development Plan that provided training for farmers to increase crop yields and improve food security. A follow‐up survey three years after the plant opened found that most health indicators had improved as a result of these measures (Knoblauch et al. 2014).

**TABLE 3:** Health impacts identified in HIA of a commercial biofuel facility in Sierra Leone (Manley et al. 2011).

Pathways	Health impacts
Employment	Increased incomes leading to reduced malnutrition Occupational injuries
Diversion of agricultural land to grow sugar cane for biofuel	Reduced food production leading to increased malnutrition
Clearing and cultivation of land	Exposure to rodents leading to risk of Lassa fever Air pollution Irrigation increasing mosquito breeding sites leading to increased malaria
Increased road infrastructure and traffic	Increased road injuries Air pollution
In‐migration	Crowding and housing insecurity Pressure on sanitation leading to infectious diseases Pressure on healthcare services leading to poorer care Spread of sexually transmitted infections

### 4.2 Changes in Healthcare Delivery

Introduction of new technologies in healthcare often requires changes in service design and delivery, which may have wider impacts for staff and patients. One of the authors (LG) facilitated a rapid HIA of the redesign of microbiology services in North Wales, involving a stakeholder workshop to identify the potential impacts and inform recommendations. The redesign created new working patterns for staff and was prompted by investments in new technology that required centralisation of services. Table 4 summarises the impacts identified in the workshop. In this case there were relatively few implications for patients and most of the impacts were for staff affected by changes in work location and working patterns. The workshop also identified that some groups of staff would be more affected because of personal circumstances such as caring responsibilities or geographical distance from the new base. In this example, the workshop was sufficient to raise these impacts, and no further evidence was gathered for the assessment. The workshop participants made recommendations to mitigate negative impacts and realise the opportunities provided by the new service model. These included increasing the numbers of staff involved in the new working patterns to reduce the pressures on overtime and on‐call duties, actions to reduce noise in the new, larger site and ongoing staff support to respond to individual needs.

**TABLE 4:** Health Impacts identified in HIA workshop on redesign of microbiology services.

Area of impact	Identified impacts
Social and community influences	Impact on family relationships including childcare Loss of sense of belonging as teams are integrated into larger service Lack of space for informal connections in new building
Living/environmental conditions	Better facilities in new building creates better working environment Noise and poor lighting in new building Increased travel distances for staff with concerns about personal safety travelling after dark
Economic conditions	Reduced income due to changes in on call payments Potential career development opportunities with centralised team
Health related behaviour	Lack of suitable space for staff to eat lunch and ‘fast food’ options for working late may adversely affect diet Shift work may limit opportunities for physical activity
Access and quality of services	Centralised service means samples transported further, which may increase time to provide results Improved opportunities for staff training and development

These examples show a range of impacts on health, well‐being and equity identified for these contrasting developments. They demonstrate that the kinds of impacts vary by context and the characteristics of affected people, as well as the specific features of the proposal being assessed. The scope and scale of assessment should also be proportionate to the scale of the project and its likely importance for health. The impacts identified in these examples are by no means comprehensive of all the impacts that may be relevant for different kinds of microbial biotechnology in different contexts.

## 5 Can HiAP Prevent Microbial Biotechnologies Being Used for Harm?

Some microbial biotechnologies, such as gain of function technology, may be perceived to carry significant inherent risks (Casadevall et al. 2024; Duprex et al. 2015). Others may have the potential to be misused or subverted for other purposes by rogue actors (Klietmann and Ruoff 2001), and some may be used deliberately to harm human health, for example in military applications (Armstrong 2017; Timmis 2001). Once a biotechnology is known it is likely to be used and built upon elsewhere, including any uses that could be harmful. HiAP could in theory help to reduce the risk of biotechnologies being subverted for harmful purposes if used to identify these risks prospectively and influence the regulatory context in which new biotechnologies are developed and shaped. However this raises multiple challenges. Firstly, it would need to be applied at a very early stage in the development of new applications so actions could be taken to prevent or manage uses that are harmful to health. Currently, HIAs are usually applied at a later stage, to proposed policies and plans that are well defined, have a clear purpose and provide sufficient detail to undertake an assessment (Winkler et al. 2021). Secondly, development of biotechnologies is often undertaken in the private sector or otherwise covered by secrecy laws, making it more difficult to assess their benefits and risks to health (Dedeurwaerdere 2010). Thirdly, there would be a significant evidence gap that would limit any assessment of the health impacts of applications that have not even been developed yet—it implies identifying and assessing ‘unknown unknowns’. There is a need for a global governance mechanism to oversee the development and introduction of new microbial biotechnologies to reduce these risks (Dietz et al. 2023; Newell and Mackenzie 2004).

## 6 How Can Understanding of Microbial Biotechnologies Inform and Contribute to HiAP?

An understanding of microbial biotechnology may also inform HIAs. There are some examples of HIAs of proposals for water and sanitation infrastructure that have used microbial sampling in the appraisal step of HIA (Hargrove and Juarez‐Carillo 2014; Seaton and Sinclair 2019). These identified pathogen contamination of soil and water, informing priorities for improvements. Knowledge of microbial biotechnologies may also inform the recommendations step of HIA by suggesting measures to mitigate some of the negative impacts and enhance some of the positive impacts identified. For example, HIAs of infrastructure projects often identify impacts arising from the influx of workers. One potential impact from this is disruption of traditional patterns of food production and distribution, leading to food insecurity amongst vulnerable populations (International Council on Mining and Metals 2010). A potential measure to mitigate this could be the deployment of microbial biotechnologies that improve soil health, increase crop plant yields, and hence improve food security (Gao et al. 2022; Maestre et al. 2017). An HIA of some industrial developments with high water usage may identify the potential for reduced availability of water for drinking and irrigation with substantial negative health impacts. A potential mitigation measure could be water purification technology that enables recycling of industrial process waters to reduce water usage (Lovett 2024).

## 7 Discussion and Conclusions

Application of new technologies across many fields can affect multiple outcomes beyond their intended direct effects, including economic, social and environmental impacts. All of these may affect health, often through indirect pathways. Other authors have argued for Health in All Policies approaches as a way to ensure governance of clinical diagnostic technologies to ensure that these wider impacts are considered (Dove et al. 2015). Similar considerations apply to microbial biotechnologies. This paper has discussed some of the ways in which the development and use of microbial biotechnologies can affect multiple determinants of health and how Health in All Policies approaches such as HIA could help to ensure that they are implemented in ways that maximise benefits and reduce any risks to health. HIA has been applied in many sectors (Lamprecht et al. 2024), but we found few publicly available examples of its application to microbial biotechnologies. The many potential applications of microbial biotechnologies in the food‐agricultural, energy, mining, environment, materials and packaging, clothing‐fashion, economic‐employment sectors could have a wide range of health impacts that should be recognised and assessed. As more biotechnologies are developed and implemented, more routine use of HIA could help them avoid adverse effects and realise the potential to improve health and reduce health inequalities.

Many important health impacts are not directly related to the specific biotechnology but depend on how it is developed and used, who owns the rights to its development and use and how technical, financial and other benefits are distributed (Dedeurwaerdere 2010). A Health in All Policies approach promotes health equity by highlighting these distributional effects so that action can be taken to ensure benefits are directed to the populations most in need (Green et al. 2021; Heller, Malekafzali, et al. 2014). Sometimes introduction of new biotechnologies with important potential direct health benefits can be contested (McKie 2024). This resistance may be for many reasons including concerns that affect health or health determinants in other ways. These could include, for example, concerns about the need for change to traditional practices and livelihoods or the pschosocial impacts of ‘solastalgia’ which is distress caused by unwanted environmental change (Albrecht et al. 2007). HIA is not intended as a way to reduce conflict but may help to engage stakeholders, identify the concerns and can make explicit both the benefits and any harms, and who will bear these (Public Health Scotland 2024).

So what is needed to adopt a Health in All Policies approach to the development, introduction and use of microbial biotechnologies? Greater awareness and understanding of the breadth of links to health and the concept of Health in All Policies is an important step, and further research may be needed to provide the evidence base for some of these links. There is also a need for governance mechanisms to require full consideration of the health impacts. Financial institutions require funded industrial and infrastructure projects to meet performance standards for occupational and community health and safety (Equator Principles 2025; International Finance Corporation 2012). HIA is used to meet some of these requirements (International Finance Corporation 2009). Similar governance standards and requirements should be applied to the development and implementation of microbial biotechnologies. Adopting a Health in All Policies approach within these mechanisms could ensure microbial biotechnologies are designed and used to meet all the SDGs and promote health, equity and sustainability.

## Author Contributions

**Margaret J. Douglas:** conceptualization, writing – original draft, writing – review and editing, investigation. **Liz Green:** writing – review and editing. **James Timmis:** writing – review and editing, conceptualization. **Timo Clemens:** supervision, writing – review and editing. **Kenneth Timmis:** conceptualization, writing – review and editing.

## Conflicts of Interest

The authors declare no conflicts of interest.

## Data Availability

Data sharing is not applicable to this article as no new data were created or analyzed in this study.
